# Long-term trends in pancreatic cancer mortality in Spain (1952–2012)

**DOI:** 10.1186/s12885-018-4494-3

**Published:** 2018-06-04

**Authors:** Daniel Seoane-Mato, Olivier Nuñez, Nerea Fernández-de-Larrea, Beatriz Pérez-Gómez, Marina Pollán, Gonzalo López-Abente, Nuria Aragonés

**Affiliations:** 1 Research Unit, Spanish Society of Rheumatology, Madrid, Spain; 2 Cancer and Environmental Epidemiology Unit, National Center for Epidemiology, Carlos III Institute of Health, Madrid, Spain; 3 Consortium for Biomedical Research in Epidemiology and Public Health (CIBER Epidemiología y Salud Pública, CIBERESP), Madrid, Spain

**Keywords:** Pancreatic cancer, Tobacco smoking, Mortality, Age-period-cohort analysis, Change-points, Time trends, Spain

## Abstract

**Background:**

Pancreatic cancer is acquiring increasing prominence as a cause of cancer death in the population. The purpose of this study was to analyze long-term pancreatic cancer mortality trends in Spain and evaluate the independent effects of age, death period and birth cohort on these trends.

**Methods:**

Population and mortality data for the period 1952–2012 were obtained from the Spanish National Statistics Institute. Pancreatic cancer deaths were identified using the International Classification of Diseases ICD-6 to ICD-9 (157 code) and ICD-10 (C25 code). Age-specific and age-adjusted mortality rates were computed by sex, region and five-year period. Changes in pancreatic cancer mortality trends were evaluated using joinpoint regression analyses by sex and region. Age-period-cohort log-linear models were fitted separately for each sex, and segmented regression models were used to detect changes in period- and cohort-effect curvatures.

**Results:**

In men, rates increased by 4.1% per annum from 1975 until the mid-1980s and by 1.1% thereafter. In women, there was an increase of 3.6% per annum until the late 1980s, and 1.4% per annum from 1987 to 2012. With reference to the cohort effects, there was an increase in mortality until the generations born in the 1950s in men and a subsequent decline detected by the change point in 1960. A similar trend was observed in women, but the change point occurred 10 years later than in men.

**Conclusions:**

Pancreatic cancer mortality increased over the study period in both sexes and all regions. An important rise in rates -around 4% annually- was registered until the 1980s, and upward trends were more moderate subsequently. The differences among sexes in trends in younger generations may be linked to different past prevalence of exposure to some risk factors, particularly tobacco, which underwent an earlier decrease in men than in women.

**Electronic supplementary material:**

The online version of this article (10.1186/s12885-018-4494-3) contains supplementary material, which is available to authorized users.

## Background

Though, in terms of incidence, pancreatic cancer is not among the most frequent cancers, its high lethality places this malignant tumor among those that cause a higher number of deaths worldwide [1]. The overall prognosis of pancreatic cancer is extremely poor, with five-year relative survival rates around 6% in Europe [2].

In Spain, 6367 new pancreatic cancer cases were estimated to occur in 2012, with age adjusted incidence rates (European standard population) of 11.5 cases per 100,000 males and 7.6 cases per 100,000 females [1]. According to these data, this cancer has become the tenth most common cancer type registered among men and the sixth among women.

As regards to mortality, in 2012 pancreatic cancer ranked seventh as cause of cancer death among Spanish men, with age adjusted mortality rates of 10.7 per 100,000 inhabitants [3], being the third leading cause of oncologic deaths among men between 40 and 59 years [4]. In women, pancreatic cancer was the fourth most common cause of oncologic death, with rates around 6.8 per 100,000. In sum, pancreatic cancer is currently responsible for 5 and 7% of the total number of deaths due to cancer among Spanish males and females, respectively.

The etiology of pancreatic cancer is unclear [5] and, as in other cancers, probably multifactorial. Several factors have been suggested as possible causes for this neoplasm, but their contribution, according to their relative risks, is small [6]. Between 3 and 7% of cases could be associated with genetic susceptibility. Regarding exogenous exposures, tobacco smoking (with strong evidence) and *Helicobacter pylori* infection (with moderate evidence) have been considered the major risk factors for pancreatic cancer, in terms of their population attributable fraction, in a recent review [6]. According to the American Institute for Cancer Research, there is also convincing evidence to consider body fatness as a risk factor for pancreatic cancer [7]. Other exposures or clinical entities that have been suggested to be associated with increased risk include high red meat consumption, type II Diabetes Mellitus, chronic pancreatitis, high alcohol consumption, hepatitis B virus infection and specific occupational exposures, such as certain pesticides, organic solvents, polycyclic aromatic hydrocarbons and nickel compounds [6–9]. On the other hand, several factors could have a preventive effect, as it is the case of allergic conditions, high fruit and vegetable consumption and physical activity [6–8].

This study aimed to monitor pancreatic cancer mortality trends since the middle of the twentieth century until recent years in Spain, using joinpoint regression models and age-period-cohort analyses to evaluate the independent effects of age, death period and birth cohort on these trends.

## Methods

Mortality data for the calendar period 1952–2012 were obtained from the Spanish National Statistics Institute (*Instituto Nacional de Estadística*) at national level. During this period, different revisions of the International Classification of Diseases (ICD) have been used. Codes selected to identify deaths due to pancreatic cancer were adapted accordingly: code 157 in the ICD-6 to ICD-9 and code C25 in the ICD-10. Population data corresponding to censuses and municipal rolls for the midyear of each quinquennium were also obtained from the Spanish National Statistics Institute.

From 1975 to 2012, mortality and population data are public and available at regional level, and were stratified by sex, five-year-age group (from 0 to 4 to 85+ years), calendar year and region (Autonomous Community). Age-adjusted mortality rates (AAMR) per 100,000 person-years were then calculated, by the direct method, for each sex, five-year calendar period and region, using the 1976 European Standard Population (ESP). Age-adjusted mortality rates were also calculated using the 2013 ESP to allow comparison with other works (Additional files 1 and 2) [10]. Additionally, annual age-adjusted mortality rates and their corresponding standard errors were calculated to study time trends for each region and sex. We used the joinpoint regression analysis to evaluate the presence of change points in adjusted mortality rates over time by sex and region and to estimate the annual percent of change over the study period [11]. Ceuta and Melilla regions were excluded from this analysis, because of their small populations.

With respect to age-period-cohort models, first, age-specific mortality rates per 100,000 person-years were computed by sex and calendar period (using five-year periods) at the national level. Then, separate log-linear Poisson models were fitted to study the effect of age, period of death and birth cohort for each sex on mortality trends. To address the “non-identifiability” problem (i.e. the three factors -age, period and cohort- are linearly dependent), we used Osmond and Gardner’s solution [12], as well as curvature effects and net drift as proposed by Holford [13]. The Osmond-Gardner solution splits net drift into cohort and period slopes, by minimizing any disagreement in parameter estimates between the full three-factor model and each of the two-factor models (age-period, age-cohort and period-cohort) according to their goodness of fit. Moreover, it allows to estimate two parameters not affected by the non-identifiability problem: (i) overall change over time (denominated net drift), which is the sum of the cohort and period slopes [13]; and (ii) deviation of any period or cohort estimates from the general trend (denominated curvature). Age groups < 30 years were excluded from this analysis due to the limited number of deaths. The open-ended category of persons aged 85 years and over was also excluded. We checked for extra-Poisson dispersion [14] and, where present, effects were calculated using a negative binomial distribution.

The presence of change points and 95% confidence intervals in the curvatures of the cohort and period effects was evaluated by fitting segmented models to the relationship between curvature effects and time. Details of the recursive algorithm used to estimate the segmented regression have been published elsewhere [15], and the procedure can be easily fitted using the R package “segmented” [16].

## Results

**Fig. 1:**
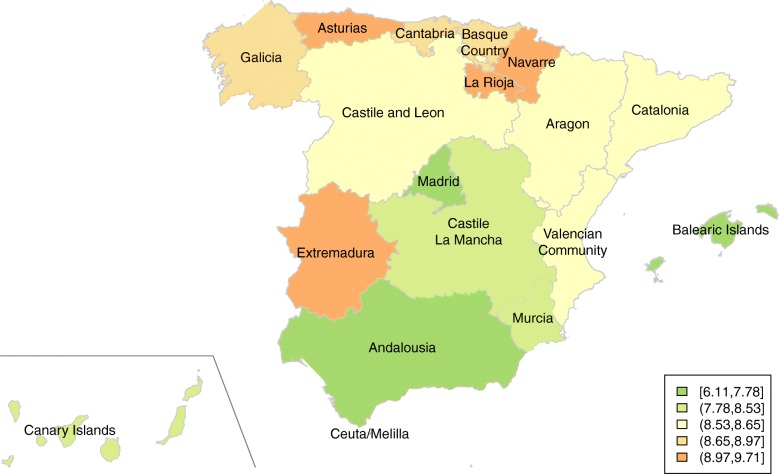
Pancreatic cancer mortality in Spain (2008–2012): AAMR per 100,000 person-years (1976 ESP) by Autonomous Community

As a geographic reference, Fig. 1 shows the location of the Spanish Autonomous Communities, with the regional distribution of pancreatic cancer mortality in both sexes in the last quinquennium (2008–2012). AAMRs (1976 ESP) by sex, Autonomous Community and calendar period are presented in Table 1. AAMRs have increased in both sexes in all regions, but not uniformly. The largest increases occurred before the 1990s in both sexes. Then, pancreatic cancer mortality rates have increased at a slower speed. No differences in this trend were observed between men and women. In both sexes, the highest increment took place between the 1978–1982 and the 1983–1987 quinquennia (18% in men and 23% in women), with more modest increments thereafter (between 5 and 11%). Differences among regions over the study period have been slightly reduced: while in the quinquennium 1978–1982 the ratio between the highest and lowest rates was around 1.8 in males and 1.7 in females, in 2008–2012 this ratio was around 1.3 in both sexes. In men, the highest rates in the quinquennium 2008–2012 were found in Asturias, La Rioja and Galicia, whereas in women the highest rates corresponded to Navarra, Asturias and Cantabria. Since a new European Standard Population has been published recently, AAMRs were recalculated using the 2013 ESP (Additional file 1: Figure S1 and Additional file 2: Table S1). Results are similar, though AAMRs tend to be higher when using the 2013 ESP, given the greater weight that this new standard population gives to older age groups.

**Table 1:** Pancreatic cancer mortality in Spain (1978–2012) by sex, Autonomous Community and calendar period^a^

		1978–82	1983–87	1988–92	1993–97	1998–02	2003–07	2008–12
Men	Andalucía	5.34	6.18	6.71	7.37	7.57	8.29	9.34
	Aragón	5.91	7.57	9.39	8.95	9.83	10.38	11.17
	Asturias	8.81	11.67	10.26	9.79	11.10	11.64	12.37
	C.Valenciana	6.33	7.84	7.71	8.45	9.33	9.04	10.60
	Cantabria	7.88	9.49	10.78	10.74	10.33	11.79	10.81
	Castilla y León	6.43	7.36	8.39	9.38	9.06	10.46	10.59
	Castilla-la Mancha	4.86	5.96	6.78	7.18	7.74	8.68	9.26
	Cataluña	6.91	8.34	8.95	9.67	9.44	10.07	10.79
	Ceuta	5.14	10.31	11.69	14.56	13.21	13.66	9.54
	Extremadura	6.18	6.86	9.18	8.86	10.50	9.80	11.36
	Galicia	5.90	8.14	8.54	9.70	10.85	10.99	11.47
	Islas Baleares	6.57	7.89	9.17	7.99	9.38	9.81	9.57
	Islas Canarias	9.26	9.30	10.77	9.66	10.38	10.55	10.21
	La Rioja	7.77	8.01	10.43	9.67	9.08	10.46	12.17
	Madrid	5.93	5.76	7.99	7.92	9.31	9.52	9.19
	Melilla	1.68	2.09	0.87	3.77	8.49	8.34	8.39
	Murcia	6.16	6.33	6.48	7.73	9.06	9.66	10.21
	Navarra	6.49	8.25	9.89	10.98	11.37	10.87	11.30
	País Vasco	7.28	8.89	9.49	10.74	10.42	10.65	10.75
	Spain	6.33	7.49	8.31	8.81	9.32	9.77	10.32
Women	Andalucía	3.41	4.11	4.37	4.75	4.72	5.02	5.97
	Aragón	3.83	4.85	5.14	6.01	5.87	6.33	6.37
	Asturias	5.27	6.51	5.80	6.02	6.03	6.71	7.26
	C.Valenciana	3.17	4.37	4.75	5.34	5.45	6.03	6.69
	Cantabria	4.34	5.44	6.52	8.17	5.97	7.19	7.14
	Castilla y León	3.57	4.61	4.74	5.35	5.69	5.77	6.81
	Castilla-la Mancha	3.12	3.75	4.87	4.69	4.89	5.75	6.53
	Cataluña	3.97	4.80	5.21	5.28	5.88	6.12	6.69
	Ceuta	6.39	7.07	7.90	6.99	8.08	6.28	4.56
	Extremadura	4.18	4.12	4.92	5.55	5.39	6.15	6.88
	Galicia	3.35	4.36	4.50	5.47	5.89	6.16	6.58
	Islas Baleares	3.52	4.44	4.95	5.77	5.58	5.98	6.01
	Islas Canarias	3.74	5.06	6.24	6.42	6.58	6.91	6.69
	La Rioja	4.46	5.57	5.53	5.44	6.95	6.27	6.61
	Madrid	3.19	3.48	4.49	5.09	5.61	5.84	6.47
	Melilla	3.45	1.25	3.63	5.18	4.28	2.97	2.46
	Murcia	4.51	3.59	4.03	5.08	4.99	5.46	6.64
	Navarra	3.86	5.28	5.12	6.49	6.69	6.97	8.18
	País Vasco	3.86	5.25	5.74	5.81	6.47	6.44	6.80
	Spain	3.64	4.46	4.89	5.36	5.61	5.96	6.57

^a^Age-adjusted mortality rates per 100,000 person-years (1976 ESP)

**Fig. 2:**
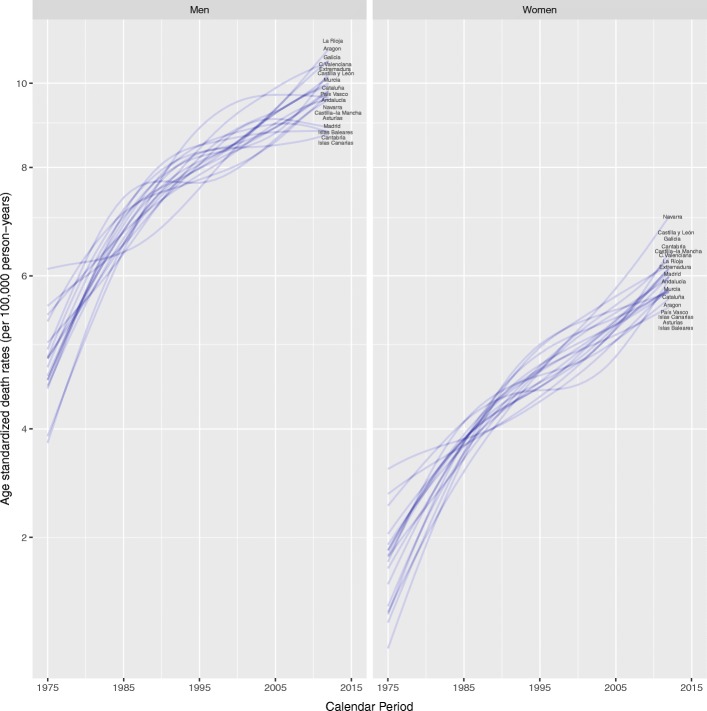
Pancreatic cancer mortality trends in Spain (1975–2012), by sex and Autonomous Community. Age-adjusted smoothed mortality rates per 100,000 person-years (1976 ESP)

The results from joinpoint regression analyses over the period 1975–2012 by Autonomous Community and sex, and in Spain as a whole are presented in Table 2. Both in men and women statistically significant upward trends were seen in nearly all Autonomous Communities. In men, there was an overall increase in the rates of 2% per annum. Joinpoint analysis detected a change point in the mid-1980s: during the first period, rates increased by 4.1% per annum and by 1.1% during the second period. In women, pancreatic cancer mortality rates experienced a marked increase of 3.6% per annum from 1975 until the late 1980s, and then increased by 1.4% per annum from 1987 to the end of the study period. By region, some of them also showed a two-phase pattern, although with differences in the year when the change was estimated to have occurred. Meanwhile, in others no inflection points were detected and rates increased at the same speed through the study period. Asturias was the Autonomous Community with the smaller overall increase (the only one under 1%) in both sexes, though their mortality rates were among the highest of the country in all quinquennia. Figure 2 shows the evolution of smoothed pancreatic cancer death rates over time by sex and region. The presence of changes in trends is visible.

**Table 2:** Pancreatic cancer mortality trend changes in Spain evaluated using joinpoint analysis by sex and Autonomous Community

				Period 1		Period 2	
		APC	N. of change points	Years	APC 1	Years	APC 2
Men	Andalucía	2.89^a^	1	1975-1978	17.66	1978-2012	1.68^a^
	Aragón	1.71^a^	0	-	-	-	-
	Asturias	0.84^a^	0	-	-	-	-
	C. Valenciana	2.14^a^	1	1975-1985	4.77^a^	1985-2012	1.19^a^
	Cantabria	0.84^a^	0	-	-	-	-
	Castilla y León	1.93^a^	1	1975-1990	3.39^a^	1990-2012	0.95^a^
	Castilla-la Mancha	2.00^a^	0	-	-	-	-
	Cataluña	1.79^a^	1	1975-1987	3.90^a^	1987-2012	0.80^a^
	Extremadura	1.80^a^	0	-	-	-	-
	Galicia	2.82^a^	1	1975-1985	6.93^a^	1985-2012	1.34^a^
	Islas Baleares	2.21^a^	1	1975-1986	6.51^a^	1986-2012	0.45
	Islas Canarias	2.37	1	1975-1978	29.02	1978-2012	0.30
	La Rioja	1.38^a^	0	-	-	-	-
	Madrid	1.62^a^	1	1975-2001	2.50^a^	2001-2012	-0.42
	Murcia	1.74^a^	0	-	-	-	-
	Navarra	1.39^a^	0	-	-	-	-
	País Vasco	1.03^a^	0	-	-	-	-
	Spain	1.99^a^	1	1975-1986	4.10^a^	1986-2012	1.11^a^
Women	Andalucía	1.57^a^	0	-	-	-	-
	Aragón	1.71^a^	0	-	-	-	-
	Asturias	0.94^a^	0	-	-	-	-
	C. Valenciana	2.78^a^	1	1975-1984	6.48^a^	1984-2012	1.61^a^
	Cantabria	2.23^a^	1	1975-1991	5.55^a^	1991-2012	-0.23
	Castilla y León	1.89^a^	0	-	-	-	-
	Castilla-la Mancha	2.34^a^	0	-	-	-	-
	Cataluña	1.52^a^	0	-	-	-	-
	Extremadura	1.65^a^	0	-	-	-	-
	Galicia	3.70^a^	1	1975-1977	38.72	1977-2012	1.99^a^
	Islas Baleares	1.54^a^	0	-	-	-	-
	Islas Canarias	1.76^a^	1	1975-1992	3.64^a^	1992-2012	0.18
	La Rioja	0.84	0	-	-	-	-
	Madrid	2.55^a^	1	1975-1994	3.61^a^	1994-2012	1.43^a^
	Murcia	1.65^a^	0	-	-	-	-
	Navarra	2.08^a^	0	-	-	-	-
	País Vasco	1.66^a^	1	1975-1989	3.03^a^	1989-2012	0.84^a^
	Spain	2.09^a^	1	1975-1987	3.56^a^	1987-2012	1.39^a^

*APC* Annual Percentage of Change

^a^Statistically significant trend as obtained from the segmented regression. Statistical tests were two sided. The significance level was considered as 0.05

**Fig. 3:**
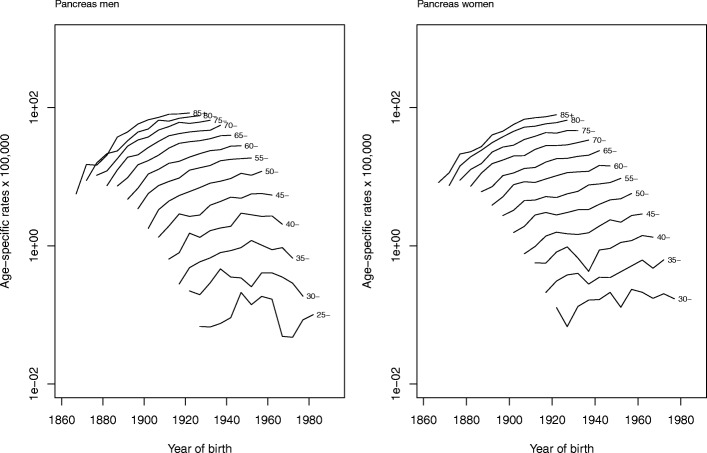
AAMR per 100,000 person-years for pancreatic cancer by birth cohort and sex, Spain (1952–2012)

Figure 3 depicts age-specific rates by birth cohort, in men and women. In both sexes, clear increases over time are observed in all age groups over 45 years of age. However, in males younger than 45 years of age a trend to stabilization, or even a reduction, is observed, while among women this trend to stabilization is only observed for the 30–35 age group. Nevertheless, trends in the youngest age groups are based on a small number of deaths.

**Fig. 4:**
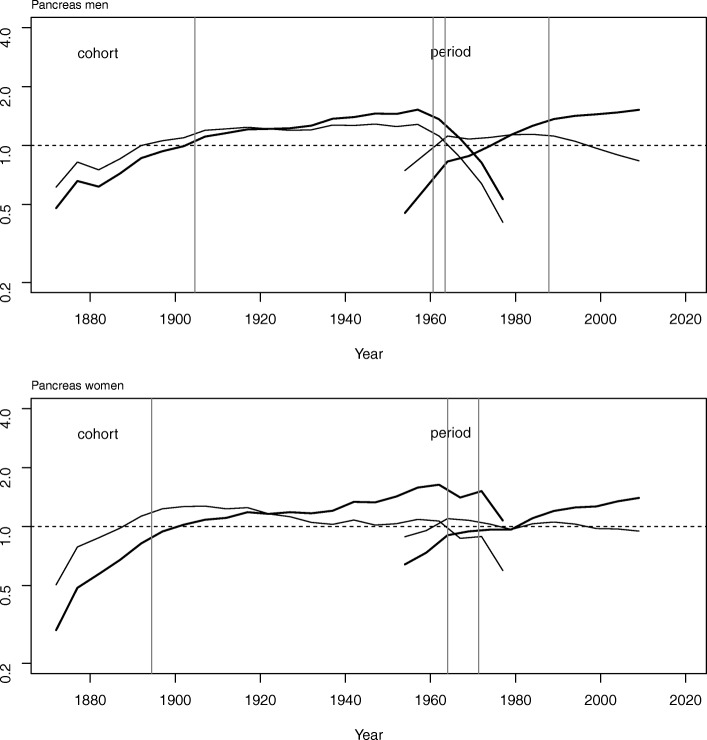
Cohort and period effects on pancreatic cancer mortality by sex, Spain (1952–2012). Cohort and period effects (thick lines), curvature (thin lines) and change points in the curvatures (vertical grey lines)

Table 3 presents the goodness of fit of the different age-period-cohort models. In men, period effect was the main contributor to the estimated trend in mortality, while in women the main contributor was the cohort effect. Figure 4 depicts cohort and period effects with the change points (listed in Table 4) detected on their curvatures. With respect to the cohort effect in men, there was an increase in mortality until the generations born in the 1950s and a subsequent decline with a detectable change point around 1960. In women, as can be seen from its flatter curvature (thin line), the cohort effect is less pronounced and the change point was placed a decade later, though this finding is difficult to assess as it is based on very few rates and deaths. For the period effect, there is a change in the general trend around 1962–1965 in both sexes, and a second change point in men around 1988, not visible in women.

**Table 3:** Goodness of fit in age, period and cohort models for pancreatic cancer mortality by sex, Spain (1952–2012)

	Degrees of freedom	Deviance	% of change in deviance
Men			
Age	121	9980	–
Age + drift	120	1382	Reference
Age + period	110	354	74.4%
Age + cohort	100	509	63.2%
Age + period + cohort	90	109	92.1%
Women			
Age	121	6094	–
Age + drift	120	694	Reference
Age + period	110	327	52.9%
Age + cohort	100	153	78.0%
Age + period + cohort	90	82	88.2%

**Table 4:** Cohort and period effect curvature change-points on pancreatic cancer mortality by sex, Spain (1952–2012)

	Changes in cohort effect^a^	
	Birth year (95% CI)	Birth year (95% CI)
Men	1904.6 (1898.7–1910.5)	1960.6 (1958.9–1962.3)
Women	1894.4 (1890.9–1897.8)	1971.2 (1968.9–1973.6)
	Changes in period effect^b^	
	Year of death (95% CI)	Year of death (95% CI)
Men	1963.5 (1961.7–1965.2)	1987.83 (1985.52–1990.14)
Women	1964.0 (1957.1–1970.9)	

^a^Year of birth with significant trend change as obtained from the segmented regression analysis of cohort curvatures from the three-factor model

^b^Year of death with significant trend change as obtained from the segmented regression analysis of period curvatures from the three-factor model

## Discussion

Our results show that pancreatic cancer mortality rates increased over the study period in Spain in both sexes and all regions. In general, this rise was similar in both sexes and in areas with higher and lower rates compared to Spain as a whole. In men, age-adjusted mortality rates annually grew on average by 4.1% in the period 1975–86, and by 1.1% between 1986 and 2012. In women there was a similar trend, with a bigger increase until the late 1980s (3.6% annually between 1975 and 1987) than in the years after (1.4% in the period 1987–2012).

The age-period-cohort analysis shows that, taking the mean rate for all cohorts as reference, the risk of dying from pancreatic cancer increased in generations born between 1870 and 1960 in men and women. This similarity in the trends could be related to changes in the exposure to risk factors linked to birth cohort and shared by both sexes. The rates observed in young men may indicate a levelling off in mortality in the most recent generations. Among women, this phenomenon is less clear, though the stabilization of the rates in the youngest age group could indicate that trend might soon parallel that of men.

For the period effect, there is a change in the general trend around 1962–1965 in males and females and a second change point in men around 1988. Though survival in pancreatic cancer is still very low, a slight improvement has been described in some countries in the last years [17–19], which could have contributed to the slower increase in the mortality rates since the 1990s observed in our study. The improvement in survival has been reported to be slightly higher in men [17]. This would be consistent with the different evolution in the period effect between men and women in the last years, with a lower annual increase of rates in men than in women since the 1990s.

In Europe, there are geographical differences in pancreatic cancer mortality trends. Mortality rates have increased in the last three decades in countries like France, Germany, Greece, Italy, Romania or Bulgaria, whereas in Sweden, the United Kingdom and Norway they have decreased (in the last two countries only in men). In Denmark, Ireland, Finland and Holland, mortality in men diminished until 1990s and then began to increase [20]. In the United States, white and black people show opposite trends: mortality rates in white men decreased from 1970 to 1995, and have increased since then; in white women, there was a slight increase between 1970 and 1984, a stabilization until the late 1990s, and an increase thereafter. On the other hand, in black men and women, rates increased until the late 1980s – early 1990s and have decreased since then [21]. In Canada, mortality rates between 1992 and 2009 declined in men and remained stable in women [22].

Understanding the reasons of the increase in pancreatic cancer mortality is challenging, given the complex and not well understood etiology of this neoplasm. In contrast with other cancer locations, like lung or cervical cancer, which are mainly associated with a unique risk factor, pancreatic cancer has been associated with multiple factors with modest effect sizes and, some of them, with high prevalence of exposure in the general population [6]. Probably, differences in latency periods among these factors may also have a different effect on pancreatic cancer trends, obscuring their specific contribution.

Among the risk factors for this cancer, the only universally accepted one is tobacco consumption [6]. Trends of pancreatic cancer mortality do not resemble those of other tumors strongly related to smoking, such as lung cancer, that is decreasing in males and increasing in females in Spain and in most developed countries. Nevertheless, the slower increase in pancreatic cancer mortality rates in the last decades could be influenced by the decreasing prevalence of tobacco consumption. In Spain, prevalence of smoking shows a decreasing trend in men since the late eighties (not previous data available), while in women, prevalence rose until the late nineties and then slightly decreased [23]. Accordingly, the continuous decrease of the rates in men cohorts born since the 1960s -not so evident in women-, could be related to the different evolution in the prevalence of smoking between sexes. Considering 2013 data, smoking prevalence in men over 34 years old had fell 9.8 percentage points in the last decade, while in women over 34 years old it had not decreased [24]. This would be consistent with a role of tobacco exposure in pancreatic cancer risk, but with the existence of other important contributing risk factors that would counterbalance the effect of the reduction in tobacco exposure.

Another suspected risk factor for pancreatic cancer is *Helicobacter pylori* infection [6]. Again, trends of pancreatic cancer mortality do not resemble those of gastric cancer, strongly associated with this infection and whose mortality rates are decreasing in both sexes. Nevertheless, according to Fig. 4, the higher increase in the risk in cohorts born until the late nineteenth century-early twentieth century and the decrease in those born since the 1960s or 1970s in men and women, respectively, is congruent with the epidemiology of *H. pylori* infection. Though this effect is not as clear as it is in gastric cancer [25], which could be explained by a weaker association between *H. pylori* infection and pancreatic cancer, there seems to be some coincidence in time between both tumors, especially in women.

Though with limited evidence, other factors have been suggested to play a role in the etiology of pancreatic cancer. Among proposed risk factors, extensive research has focused on the role of diet and anthropometric factors [26, 27]. As possible protective factors, a medical history of allergy, the consumption of fruits and vegetables, physical activity and parity have been suggested [6, 28–31]. The controversial evidence from epidemiologic studies, the diverging trends of this broad spectrum of factors and the different prevalence of exposure among regions make it not easy to disentangle the specific role of each factor in pancreatic cancer trends.

A different aspect that could have played a role in the observed upward trend in pancreatic cancer mortality is the introduction of Computerized Tomography in Spain during the late 1970s of the twentieth century, and the spreading of its use during the 1980s. In this sense, advances in diagnosis and better death certification would have yielded a rise in the period effect. However, trends become less pronounced from the 1980s onwards.

This study has some advantages and limitations that should be taken into account. Among its strengths, it involves the follow-up of the total Spanish population through 60 years. This is a dynamic cohort, with entries and exits across the study period, which encompasses generations born approximately from 1865 to 1975–1985 and thus constitutes a long time series. Also, survival of pancreatic cancer continues to be very low [2], and therefore, mortality statistics can be considered as a good proxy for incidence and are representative of the epidemiology of the disease. On the other hand, our results rely on the accuracy of death certificates and coding practices, and there could have been changes in their quality along the study period. However, cancer death certificates in Spain possess an accuracy comparable to that reported for other industrialized countries, and pancreatic cancer is among the cancer sites classified as well certified according to published quality indicators [32].

## Conclusions

This study summarizes the trends in pancreatic cancer mortality in Spain, allowing for a detailed analysis of the influence of age, period and cohort effects in each sex. Like in other developed countries, pancreatic cancer mortality has been increasing over the last decades. However, differences have been identified between sexes. In men, mortality rates show a stabilization or even a decrease since the early 1990s, mainly among post-1960 birth cohorts, while in women, a stabilization in the trend is observable only among the youngest generations (born after the late 1970s). These differences may partially mirror the evolution of some established risk factors for pancreatic cancer, such as tobacco exposure, in men and women. Further research on the causes of pancreatic cancer is needed. In the meanwhile, recommendations to reduce exposure to preventable risk factors that have been associated with pancreatic cancer, such as tobacco smoking, obesity, diet and lack of physical activity may serve to reduce the impact of this and other chronic and malignant diseases.

## Additional files

Additional file 1 **Figure S1.** Pancreatic cancer mortality in Spain (2008–2012): AAMR per 100,000 person-years (2013 ESP) by Autonomous Community. (PDF 529 kb)

Additional file 2 **Table S1.** Pancreatic cancer mortality in Spain. AAMR per 100,000 person-years (2013 ESP) by sex, Autonomous Community and calendar period. (DOCX 30 kb)

## Abbreviations

AAMR: Age-adjusted mortality rates
ESP: European Standard Population
ICD: International Classification of Diseases
