# Supplementary figures and images for: Long-term trends in pancreatic cancer mortality in Spain (1952–2012)
Source: BMC Cancer. 2018 Jun 4;18:625. doi: 10.1186/s12885-018-4494-3 (PMC5987643; doi:10.1186/s12885-018-4494-3)

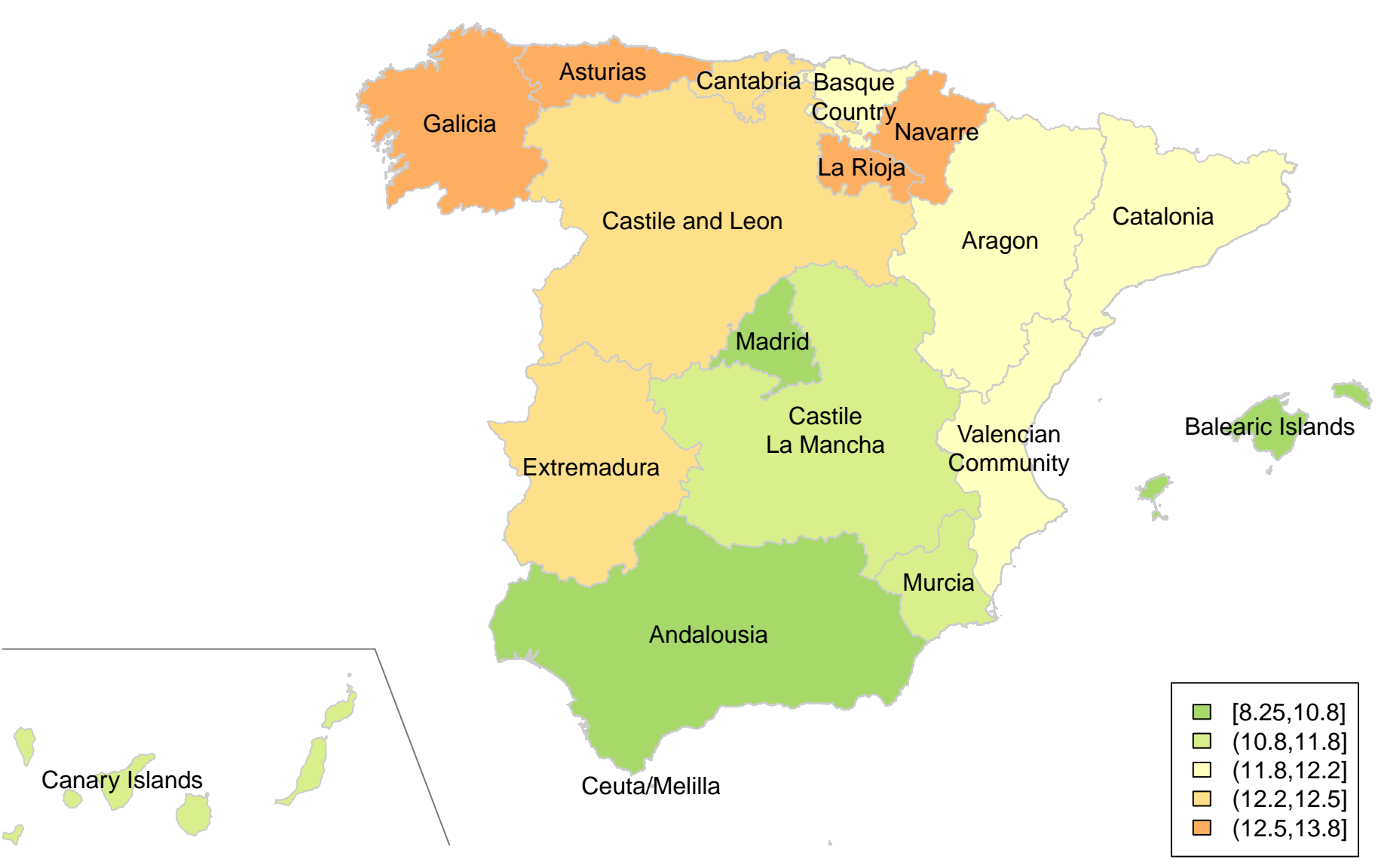

Supplement: Supplementary file 1 — Figure S1. Pancreatic cancer mortality in Spain (2008–2012): AAMR per 100,000 person-years (2013 ESP) by Autonomous Community. (PDF 529 kb) [file 12885_2018_4494_MOESM1_ESM.pdf]
