# Supplementary material for: Long-term trends in pancreatic cancer mortality in Spain (1952–2012)
Source: BMC Cancer. 2018 Jun 4;18:625. doi: 10.1186/s12885-018-4494-3 (PMC5987643; doi:10.1186/s12885-018-4494-3)
Supplement: Supplementary file 2 — Table S1. Pancreatic cancer mortality in Spain. AAMR per 100,000 person-years (2013 ESP) by sex, Autonomous Community and calendar period. (DOCX 30 kb) [file 12885_2018_4494_MOESM2_ESM.docx]

| Table S1. Pancreatic cancer mortality in Spain by sex, Autonomous Community and calendar period.* | | | | | | | | |
| --- | --- | --- | --- | --- | --- | --- | --- | --- |
|  |  | **1978-82** | **1983-87** | **1988-92** | **1993-97** | **1998-02** | **2003-07** | **2008-12** |
|  |  |  |  |  |  |  |  |  |
| **Men** | Andalucía | 7.26 | 8.51 | 9.26 | 10.23 | 10.40 | 11.28 | 13.06 |
|  | Aragón | 8.37 | 10.03 | 12.92 | 12.31 | 13.11 | 14.63 | 15.81 |
|  | Asturias | 12.32 | 16.49 | 14.42 | 13.08 | 14.85 | 15.44 | 16.77 |
|  | C.Valenciana | 8.54 | 11.06 | 10.76 | 11.66 | 12.99 | 12.44 | 14.69 |
|  | Cantabria | 10.79 | 12.21 | 14.96 | 14.93 | 13.53 | 16.27 | 14.87 |
|  | Castilla y León | 8.76 | 9.96 | 11.49 | 12.60 | 12.52 | 14.29 | 14.83 |
|  | Castilla-la Mancha | 6.53 | 8.27 | 9.30 | 10.25 | 10.57 | 11.96 | 12.89 |
|  | Cataluña | 9.45 | 11.54 | 12.24 | 13.18 | 12.98 | 13.95 | 14.96 |
|  | Ceuta | 8.49 | 11.17 | 16.56 | 22.17 | 17.70 | 19.93 | 11.93 |
|  | Extremadura | 8.79 | 9.19 | 12.40 | 12.28 | 14.63 | 13.70 | 15.76 |
|  | Galicia | 8.27 | 11.34 | 11.47 | 13.49 | 14.87 | 15.35 | 16.11 |
|  | Islas Baleares | 8.96 | 11.12 | 13.37 | 11.50 | 13.02 | 13.73 | 12.98 |
|  | Islas Canarias | 13.57 | 13.13 | 15.11 | 13.78 | 14.65 | 14.78 | 13.98 |
|  | La Rioja | 10.56 | 10.72 | 14.98 | 13.87 | 11.79 | 15.13 | 17.21 |
|  | Madrid | 7.77 | 8.08 | 10.99 | 10.90 | 12.52 | 13.22 | 12.82 |
|  | Melilla | 4.19 | 3.57 | 1.19 | 5.67 | 13.55 | 11.95 | 11.54 |
|  | Murcia | 8.54 | 8.82 | 9.00 | 11.16 | 12.51 | 13.45 | 14.23 |
|  | Navarra | 9.20 | 11.50 | 13.95 | 15.64 | 16.09 | 15.31 | 16.11 |
|  | País Vasco | 10.17 | 12.57 | 13.64 | 14.81 | 13.85 | 14.74 | 15.20 |
|  | Spain | 8.69 | 10.36 | 11.48 | 12.17 | 12.75 | 13.50 | 14.38 |
|  |  |  |  |  |  |  |  |  |
| **Women** | Andalucía | 4.90 | 6.06 | 6.38 | 6.82 | 6.65 | 7.13 | 8.59 |
|  | Aragón | 5.52 | 6.95 | 7.34 | 8.77 | 8.53 | 9.03 | 8.83 |
|  | Asturias | 7.12 | 9.57 | 8.27 | 8.78 | 8.39 | 9.37 | 10.15 |
|  | C.Valenciana | 4.57 | 6.16 | 6.86 | 7.47 | 7.73 | 8.41 | 9.38 |
|  | Cantabria | 6.29 | 7.26 | 8.52 | 11.21 | 7.71 | 10.34 | 10.13 |
|  | Castilla y León | 5.32 | 6.53 | 6.78 | 7.22 | 7.91 | 8.01 | 9.74 |
|  | Castilla-la Mancha | 4.61 | 5.42 | 6.85 | 6.58 | 6.70 | 7.98 | 9.05 |
|  | Cataluña | 5.64 | 6.71 | 7.42 | 7.37 | 8.24 | 8.66 | 9.26 |
|  | Ceuta | 7.96 | 10.20 | 10.13 | 10.51 | 9.95 | 8.51 | 7.19 |
|  | Extremadura | 6.19 | 6.25 | 6.98 | 7.61 | 7.40 | 8.98 | 9.42 |
|  | Galicia | 4.79 | 6.32 | 6.43 | 7.70 | 8.34 | 8.60 | 9.41 |
|  | Islas Baleares | 5.06 | 6.56 | 6.94 | 7.94 | 7.99 | 7.97 | 8.46 |
|  | Islas Canarias | 5.53 | 7.39 | 9.15 | 9.28 | 9.32 | 9.67 | 9.41 |
|  | La Rioja | 5.76 | 8.17 | 7.78 | 7.96 | 9.32 | 9.14 | 9.86 |
|  | Madrid | 4.48 | 5.10 | 6.43 | 7.13 | 7.79 | 8.21 | 8.98 |
|  | Melilla | 4.75 | 2.51 | 4.94 | 6.51 | 6.91 | 4.39 | 2.96 |
|  | Murcia | 6.13 | 5.40 | 5.72 | 7.72 | 6.66 | 7.80 | 9.15 |
|  | Navarra | 5.84 | 7.87 | 7.60 | 9.08 | 9.81 | 9.37 | 11.64 |
|  | País Vasco | 5.33 | 7.74 | 8.36 | 8.32 | 8.98 | 8.91 | 9.53 |
|  | Spain | 5.21 | 6.43 | 7.01 | 7.55 | 7.85 | 8.36 | 9.23 |

*Age-adjusted mortality rates per 100 000 person-years (2013 European Standard Population).
